# Experimental investigation on fracturing effects in hydraulic sand fracturing with acoustic emission and 3d laser scanning

**DOI:** 10.1038/s41598-023-38775-6

**Published:** 2023-07-17

**Authors:** Shuhui Zhang, Chenghu Wang, Guangpei Zhu, Guiyun Gao, Hao Zhou

**Affiliations:** 1 Ministry of Emergency Management of China, National Institute of Natural Hazards, Beijing, 100085 China; 2 School of Energy and Mining Engineering, China University of Mining and Technology (Beijing), Beijing, 100083 China

**Keywords:** Civil engineering, Natural gas

## Abstract

Due to the extremely low permeability of shale reservoirs, large-scale reservoir fracturing is required. Hydraulic fracturing is one of the most important technologies in shale gas exploration and development. In this paper, the acoustic emission energy and the number of location and fracture surface morphology of specimens before and after fracture are studied through hydraulic sand fracturing test. The test results show that: (1) the energy ratio obtained during hydraulic fracturing without proppant is the smallest, and increasing the confining pressure, as well as reducing the displacement and viscosity of the fracturing fluid will cause the energy ratio to decrease. From the perspective of acoustic emission energy, the proppant play an important role in the generation of fractures during hydraulic sand fracturing; (2) when the confining pressure increases, the number of shale specimens before and after rupture is the largest, but the total number of locating events is smaller than the sanding ratio increased; there is no proppant hydraulic fracturing, the number of specimens before and after the rupture is the largest. And the total number reached the minimum, indicating that the proppant can play an important role in the hydraulic sand fracturing test; (3) the sand is relatively large, the specific surface and standard deviation both reach the maximum, indicating that the fracture surface roughness is the largest under the test condition, and the fracturing effect is the best, but the specific surface and standard deviation are the minimum when fracturing without proppant, so indicating that the fracture surface fracturing effect is the worst at this time.

## Introduction

Unconventional reservoir such as shale oil and gas has been an important fossil energy sources with the depletion of conventional oil and gas resources^1^. But low permeability reservoirs have been contributing a significant portion of oil and gas production. Hydraulic fracturing is the most effective reservoir-stimulation technique in the petroleum and geothermal industries^2^. It is most suitable for wells in low and moderate permeability reservoirs. Fractures created require proppant to keep it open after the injection has stopped, and the conductivity depends on the proppant distribution in the fracture^3,4^.

Cipolla et al.^5^ studied the effect of proppant distribution in the fracture network on good performance and showed that proppant distribution significantly affects the fracture network conductivity and treatment design. Tan et al.^6^ investigated the characteristics of proppant migration and distribution and their influences on the initiation and propagation of hydraulic fractures, they showed that fracture morphologies after hydraulic fracturing in coal and shale have significant differences. Palmer and Moschovidis^7^ studied the migration rules and distribution characteristics of proppants in fracture for evaluating the effective stimulated reservoir volume. Gu et al.^8^ presented that proppant transport in natural fractures has an important impact on critical fracture conductivity required for stimulation of shale reservoirs. Warpinski and Teufel^9^ showed from field results that in-situ stress was the overriding factor that influenced the fracture propagation when it was in a high-stress region compared to interfaces, modulus, strength changes, fluid pressure gradients, and most bedding planes. Stanchits et al.^10^ used acoustic emission monitoring technology to study the fracture propagation law in isotropic sandstone with the artificial interface and heterogeneous shale with the natural weak surface. Zhou et al.^11^ found that within the scope of high horizontal stress difference, hydraulic fracture was a dominating fracture with random multiple branches, while within the scope of low horizontal stress difference the hydraulic fracture was partly vertical, planar fracture with branches. Hou and Heng^12,13^ carried out shale fracturing simulation experiment and preliminarily analyzed the formation mechanism of fracture network combined with acoustic emission monitoring. Through the injection pressure curve and acoustic emission data in hydraulic fracturing test, Zhang and Li^14^ analyzed the formation mechanism of complex hydraulic fractures in tight sandstone hydraulic fracturing process.

Many scholars currently study the communication between the cracks or formation mechanism by proppant transfer or sound emission monitoring^15^. However, there is less research on the acoustic emission data and crack surface in hydraulic sand fracturing, so the paper mainly uses different stresses, fracturing fluid viscosity and displacement and sand ratio of sand fracturing test, through the analysis of acoustic emission energy, positioning events and fracture surface data, to study the difference produced under different test conditions.

## Experimental preparation

### Shale sample

Shale samples are collected from the outcrop of Scientific research well called K3, the K3 is located in Xishui County, Guizhou Province for Institute of Geology and Geophysics, Chinese Academy of Sciences special shale gas project. The mineral compositions of the shale sample mainly included quartz, white mica, cristobalite, and clay minerals, consisting of illite, montmorillonite, and albite with a small quantity of pyrite and organic matter. Quartz accounted for approximately 50.67–57.46% of the content, and the carbonate minerals calcite and albite had a negligible content and a small amount of indistinguishable amorphous material. A few clastic white micas are evident on the specimen surface. The rock mechanical strength parameters were measured by the uniaxial test: compressive strength was 136.5 MPa, elastic modulus was 24.8 GPa, and Poisson's ratio was 0.17, tensile strength 8.36 MPa.

**Figure 1:**
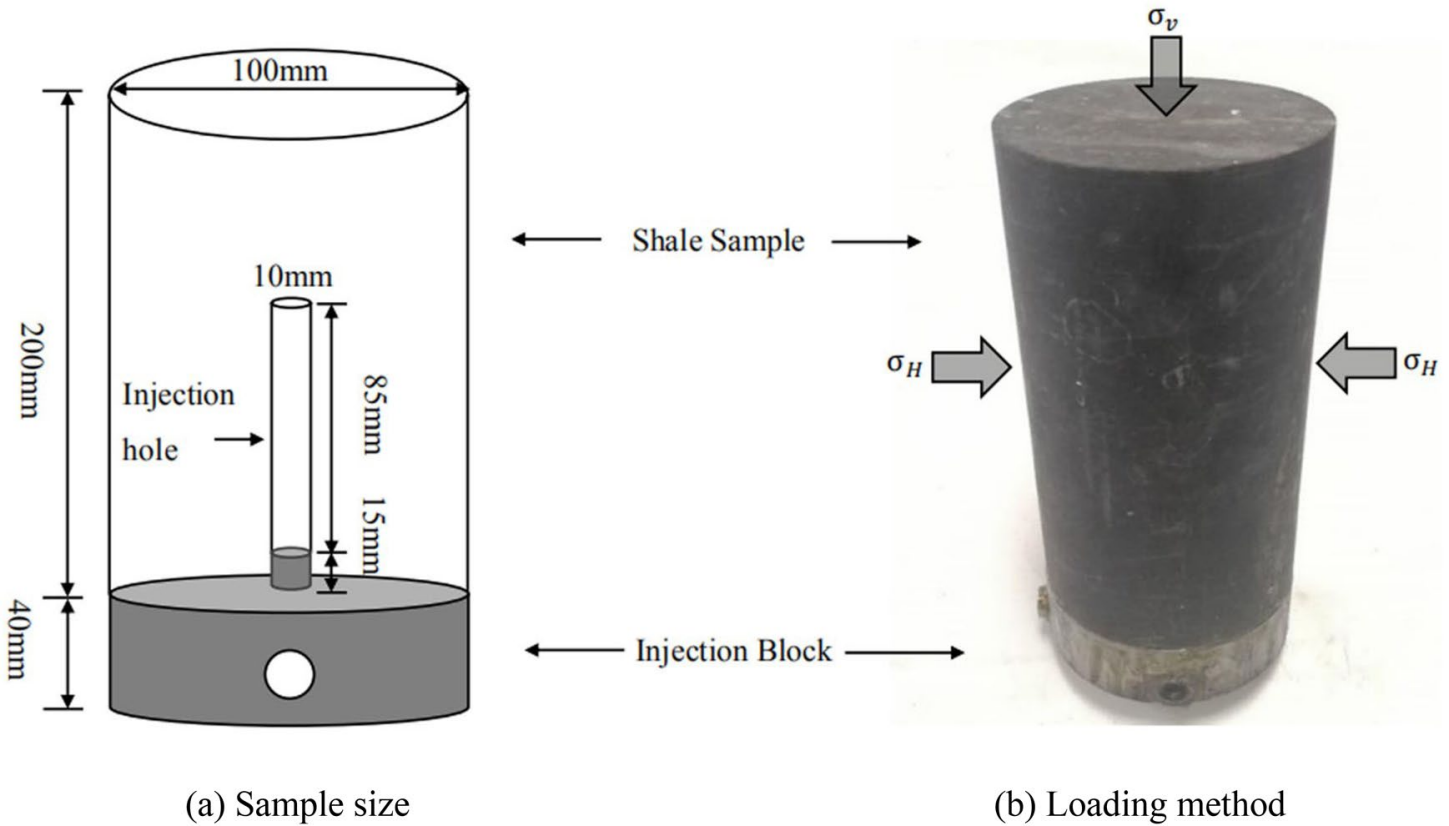
Sample design scheme^1^.

After the rock cores are obtained from the site, they are intercepted and ground into regular cylindrical samples with 100 mm in diameter and 200 mm in height. A hole with a diameter of 10 mm and a depth of 100 mm are drilled in the centers of these cylindrical samples to simulate fracturing wellbore. A 100 mm bare section is reserved inside the sample to simulate the open-hole completion. The schematic of specimen size and pressurization is shown in Fig. 1.

### Experiment design

**Figure 2:**
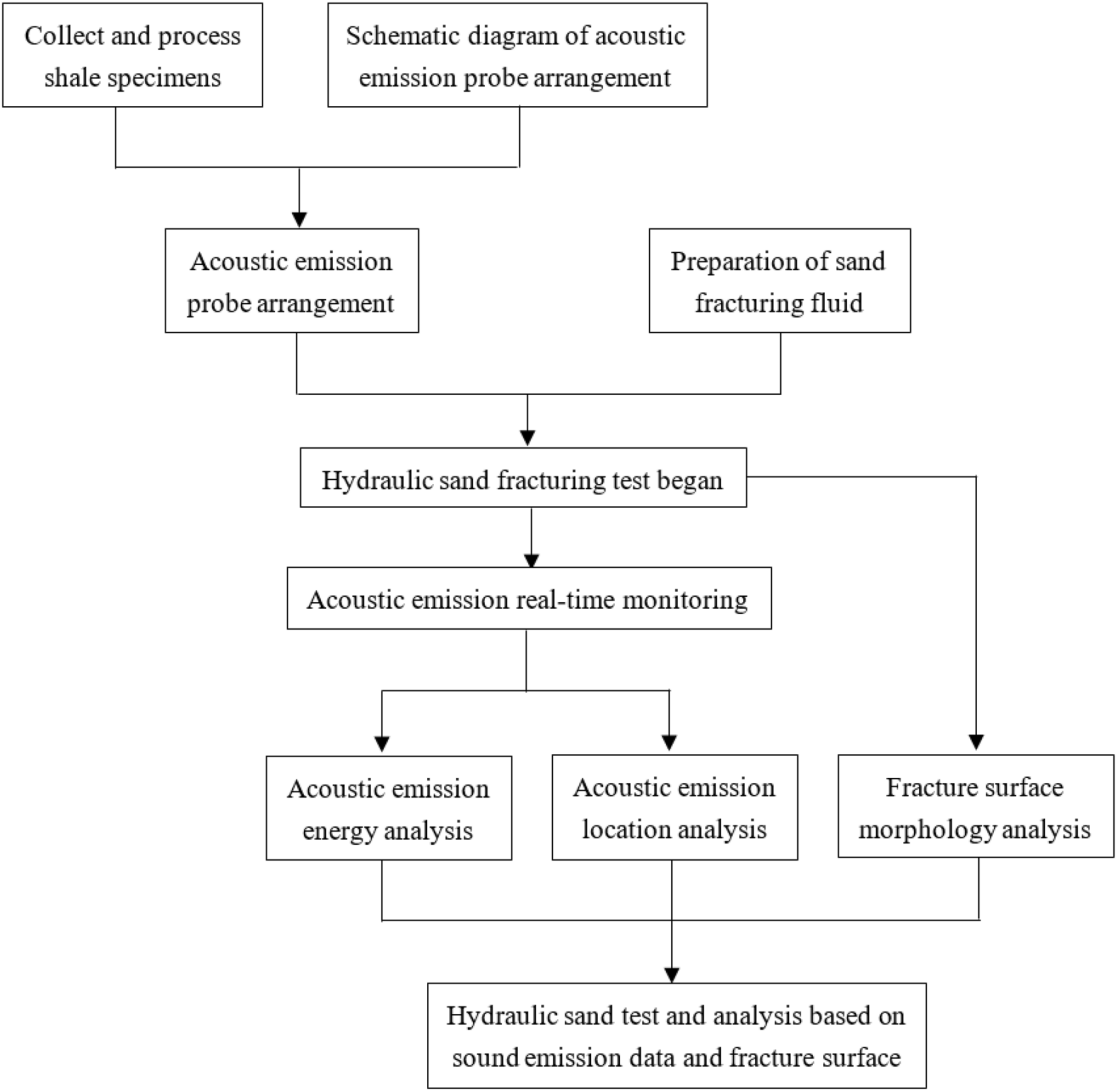
Methodology flowchart.

**Figure 3:**
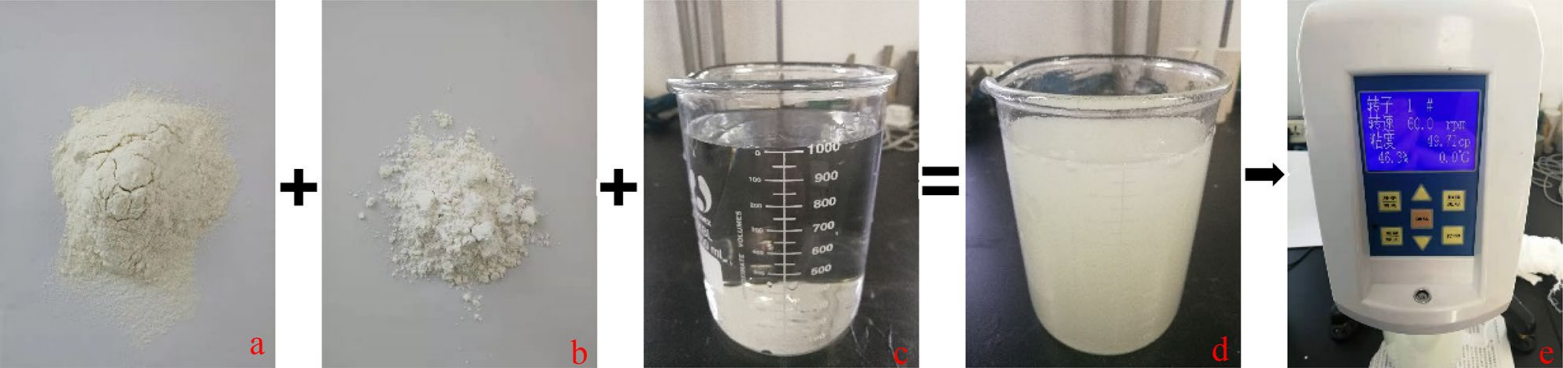
The production process of the sand fracturing fluid. a is guar gum; b is sand; c is water; d is sand fracturing fluid. e is viscosity measurement.

Due to the paper mainly studies the influence of the fracturing fluid viscosity, the fracturing test is designed to be performed under the same stress conditions. Methodology flowchart is shown in Fig. 2. According to different applied stress, fracturing fluid viscosity, injection rate and proppant content, six test pieces will be used in this paper, as shown in Table 1. The viscosity of the fracturing fluid depends on the ratio of the mass of guar and water, shown in Fig. 3. The proppant content is the ratio of the mass of quartz sand (200 mesh diameter) to the mass of water.

**Table 1:** Experiment plan.

Sample no.	Stress/MPa	Viscosity/mPa s	Rate/ml min^−1^	Sand ratio/%
G1	15/5	50	50	5
G2	15/10	50	50	5
G3	15/5	20	50	5
G4	15/5	50	20	5
G5	15/5	50	50	10
G6	15/5	50	50	0

### Sand fracturing fluid

The function of the sand fracturing fluid is to carry proppant into fractures to prevent those from closing. Sand-carrying fluid can further expand the fracture, transport and lay proppants, which is helpful in forming proppant-filling fracture with certain conductivity and designed morphology^16^. In the paper, the sand fracturing fluid is prepared by the author according to a certain proportion in the laboratory as shown in Table 2. The viscometer uses a digital rotary viscometer, the model is NDJ-5S, and the range is 1–10,000 mPa s. The configuration flow chart of the sand fracturing fluid is shown in Fig. 3.

**Table 2:** Proportion of each component in the sanding fracturing fluid.

Design viscosity/mPa s	Sand ratio/%	Actual viscosity/mPa s	Water/g	Guar gum/g	Sand/g
50	5	49.71	1000	100.35	50.82
50	10	50.31		100.35	100.20
20	5	20.85		65.42	50.23

### Acoustic emission location and moment tensor theory

**Figure 4:**
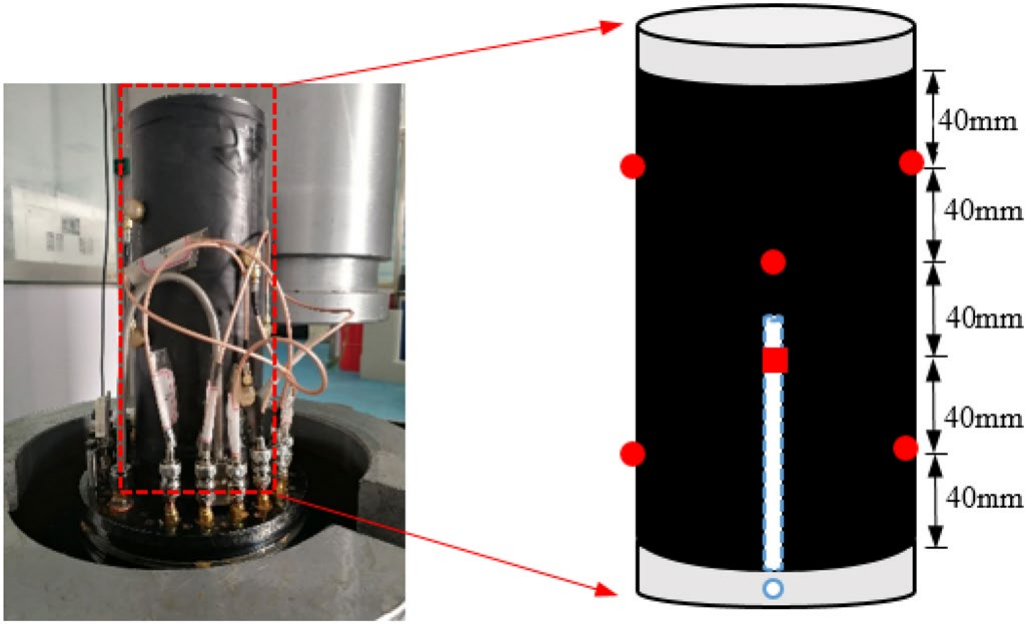
Schematic diagram of acoustic emission probe arrangement. Red circle is the sensor on the front; red square is the sensor on the back.

In order to ensure that the acoustic emission signal can be well received by the sensor, apply Vaseline to the coupling between the sensor and the test piece. In order to obtain a high-precision acoustic emission arrival time, the main amplifier of the acoustic emission test analysis system is set to 40 dB, the threshold is 45 dB, the sampling frequency is 2.5 MHz, and the sampling length is 8 k. In this paper, six special pressure sensors are used to collect acoustic emission signals. The layout is shown in Fig. 4.

## Experimental results and analysis

### Acoustic emission energy analysis of specimens

Acoustic emission energy is the sum of acoustic emission event energy received by all probes within unit sampling time. Due to matrix initiation, fracture communication and large dislocation of fracture surface, strong acoustic emission signals will be released, showing obvious high points in the acoustic emission energy curve. Therefore, acoustic emission energy analysis can be used to more accurately identify events such as fracture initiation, communication and dislocation^17^.

Since the paper is mainly to analyze the effect of proppant on fracture formation and communication during sand fracturing, the author calculates the energy collected by acoustic emission before and after fracture of shale specimen, and makes comparative analysis of different variables to study the effect of proppant on fracture formation in hydraulic sand fracturing.

**Figure 5:**
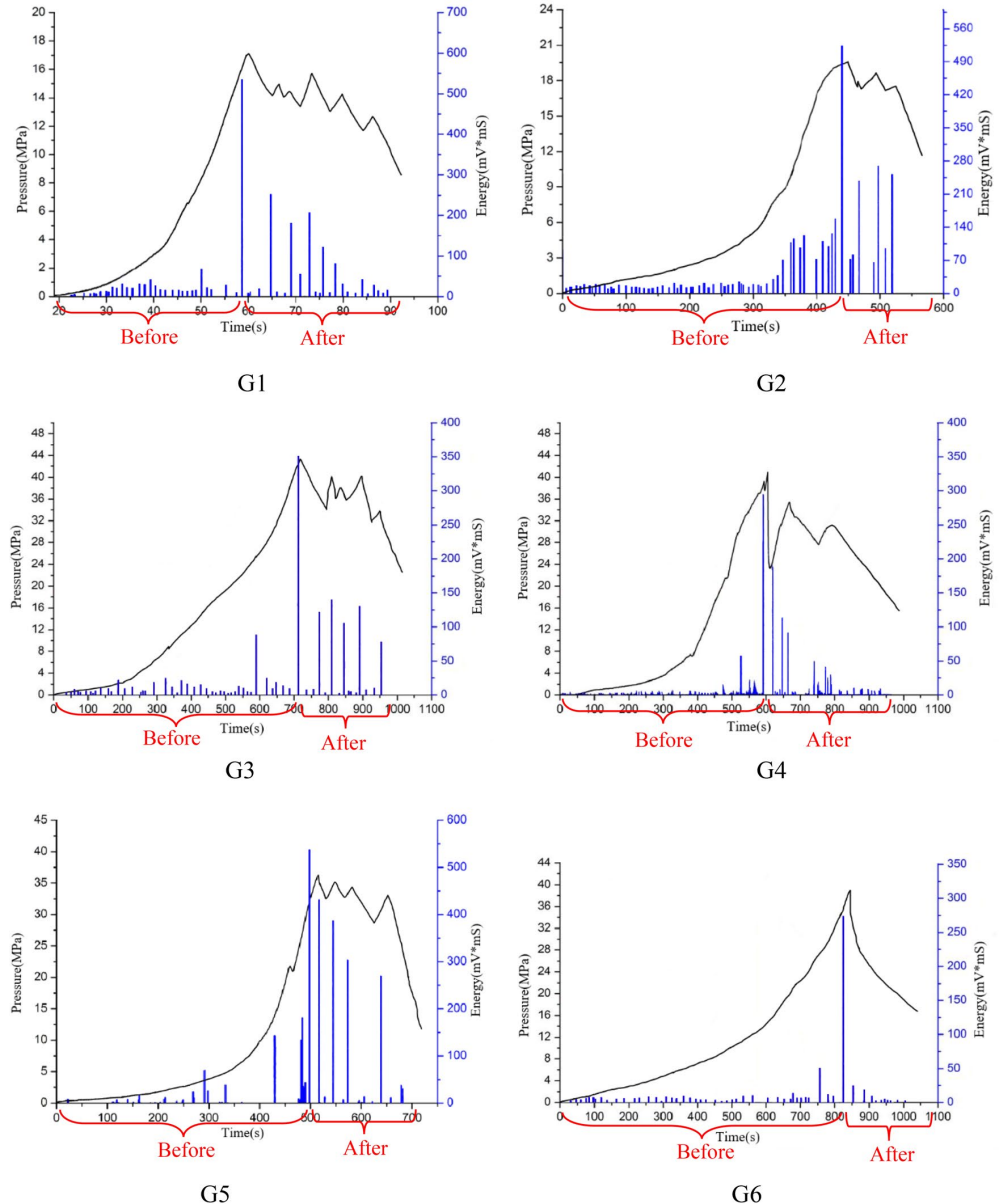
Pressure and energy-time curve.

The following conclusions can be drawn from the curves in Fig. 5. First, by comparing the energy released after fracture between G1–G5 and G6 specimens, it can be seen that proppant in hydraulic sand fracturing can expand fractures, thus forming more fractures. Second, the energy released during hydraulic sand fracturing fluctuates with the fluctuation trend of the pressure curve and shows the corresponding change trend according to different test schemes. The results are described in detail in the following paper.

Based on fracturing test time parameters from the specimen of burst fracture pressure (pressure curve vertex) to be received by acoustic emission energy is divided into two parts, before and after and for more precise quantitative analysis of proppant in specimen burst of the influence degree of the fracture, the reference ratio c were analyzed, and the rules before rupture energy summation for *Q*_1_, burst energy sum for *Q*_2_, The sum of the test energies is *Q*. *C* is defined as the ratio of the sum of energy *Q*_2_ after fracture to the sum of test energy *Q*, that is, *c* = *Q*_2_/*Q*. The value of energy collected in this test is shown in Table 3.

**Table 3:** Acoustic emission energy value.

Sample NO	*Q*_1_/(mV ms)	*Q*_2_/(mV ms)	*Q*/(mV ms)	*c*
G1	783.55	1059.38	1842.93	0.575
G2	1216.23	872.46	2088.69	0.418
G3	488.75	612.32	1101.07	0.556
G4	408.47	499.29	907.76	0.55
G5	1067.84	1891.23	2959.07	0.639
G6	338.26	52.48	390.74	0.133

**Figure 6:**
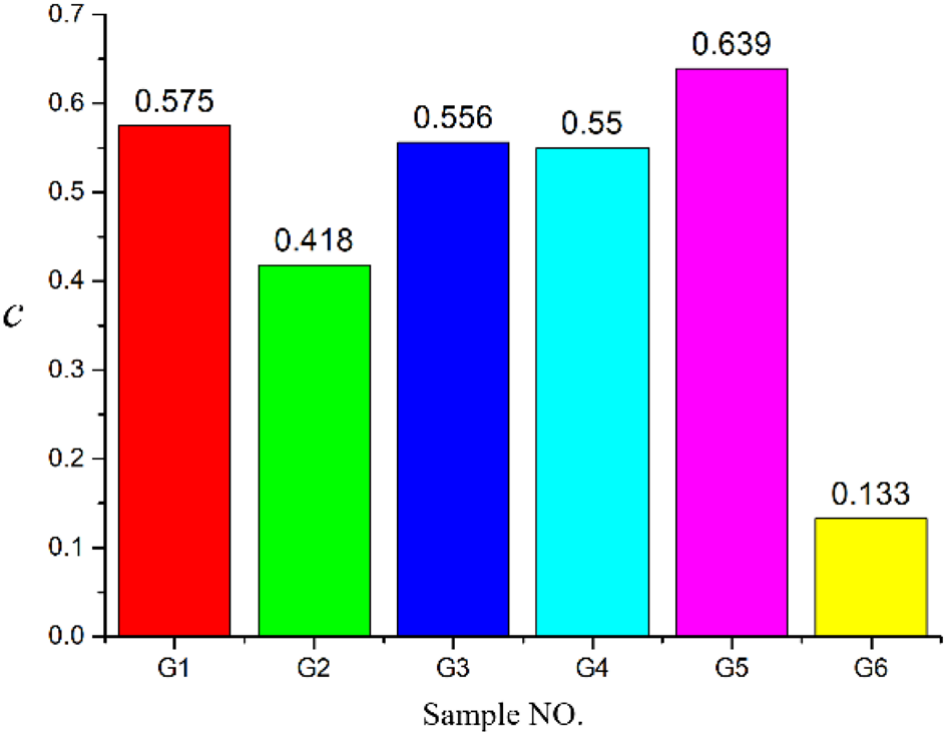
Bar graph of the energy ratio of each specimen.

Based on the energy ratio *c* of each specimen in Table 3, the summary is shown in Fig. 6. By comparing the energy values of specimens G1 and G2, G3, G4 and the ratio *c*, it can be summarized that the confining pressure, fracturing fluid viscosity and displacement The changes all affect the ability of proppant to propagate or extend fractures to a certain extent.

From the comparison of G1 and G5, it can be seen that the increase of the sand addition ratio of the fracturing fluid can increase the *Q*_1_ and *Q*_2_ and the energy ratio *c* at the same time, indicating that the increase of the sand addition ratio in the fracturing fluid can promote more proppants to enter fractures, leading to further extension or expansion of the fractures. From the energy and energy ratio *c* of the specimen G6, the fracturing fluid without proppant releases very little energy after the specimen fracture, mainly because the fracturing fluid immediately seeps out from the fracture once the specimen fracture, and the pressure varies with the pressure. If it drops, it is more difficult to reproduce crack propagation and extension.

### Analysis of acoustic emission localization event

The main definition of the number of acoustic emission events is that a local change of the material that produces acoustic emission is an acoustic emission event, and its meaning is to reflect the relevant information of the acoustic emission source, evaluate the activity of the acoustic emission source and the location of the acoustic emission source^18^. This summary is mainly to analyze the comparative evaluation of the effect of proppant adding sand under different test conditions during hydraulic sand fracturing. Therefore, the specific number of acoustic emission events before and after the rupture of the specimen is used for quantitative analysis and research.

**Figure 7:**
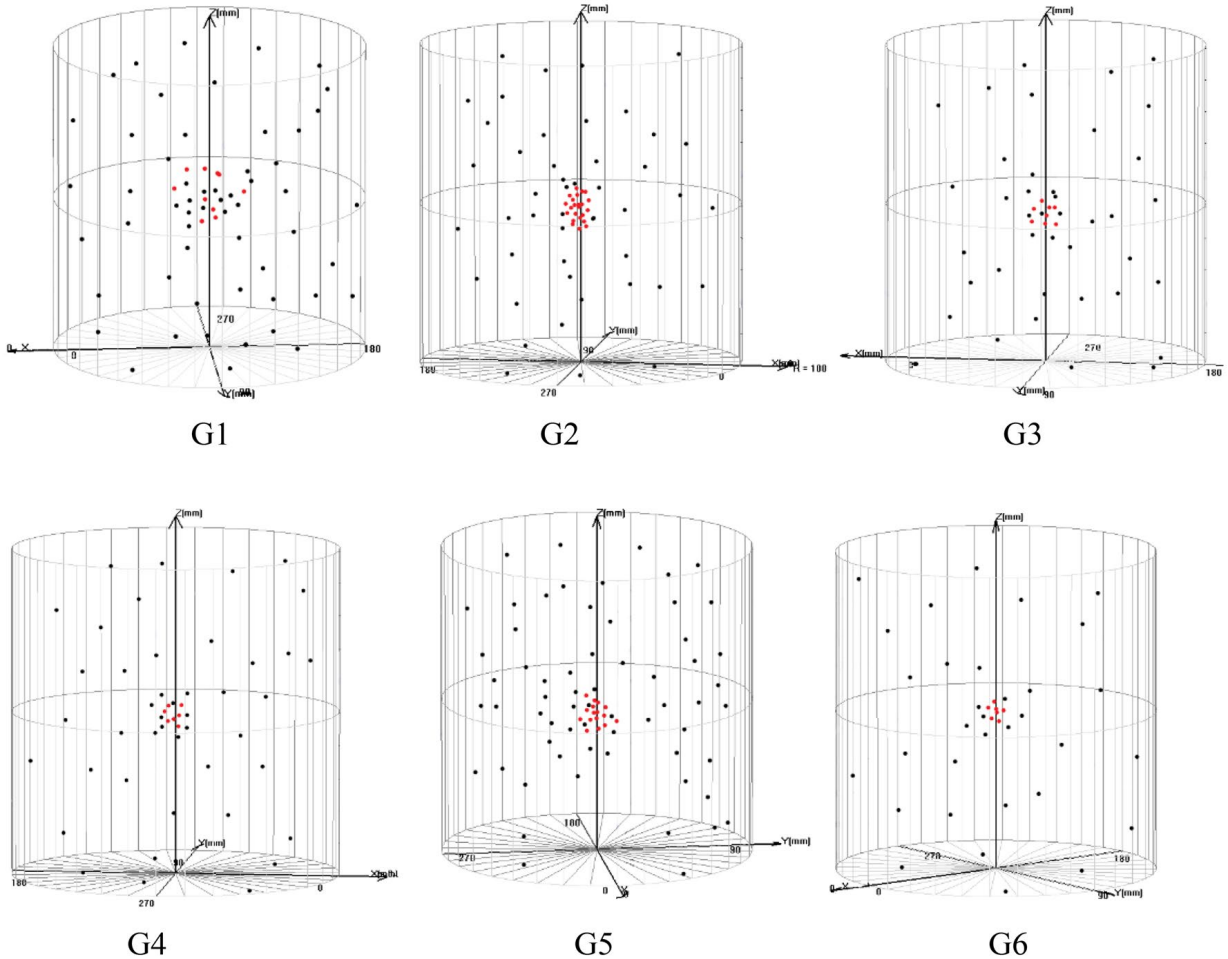
Acoustic emission location map before and after the test piece is broken. Red circle is the anchor point before the peak; black circle is the anchor point after the peak.

Counting the number of acoustic emission locations before and after the specimen fracture can be obtained as shown in Fig. 7. It can be obtained from specimens G1 and G2. Although the total number of acoustic emission locations of G2 specimens has increased to a certain extent, and the number of acoustic emissions before fracture has increased, the number of locations after fracture has decreased to a certain extent, which shows that due to the surrounding The increase in pressure caused part of the proppant to enter the fracture before the specimen ruptured, resulting in an increase in the number of acoustic emission locations, but the confining pressure was not conducive to the migration of proppant in the fracture; compare the curve data of G1, G3, and G4 to reduce the viscosity both the displacement and the displacement lead to the reduction of the number of acoustic emission positioning points before and after the specimen rupture, which indicates that the reduction of viscosity and displacement can prevent the proppant from migrating well in the fractures to produce more new fractures; the analysis of the acoustic emission location numbers of G1 and G5, it can be seen that the increase of the sand ratio can lead to better migration of the proppant before and after the fracture of the test piece, which can make more cracks propagate or extend; compare test piece G1 As with G6, it can be shown that proppant can play a greater role in fracture propagation and dislocation after fracture during hydraulic fracturing, but the role of proppant before fracture of the specimen is not particularly significant.

### Analysis of fracture surface roughness after hydraulic sand fracturing

**Figure 8:**
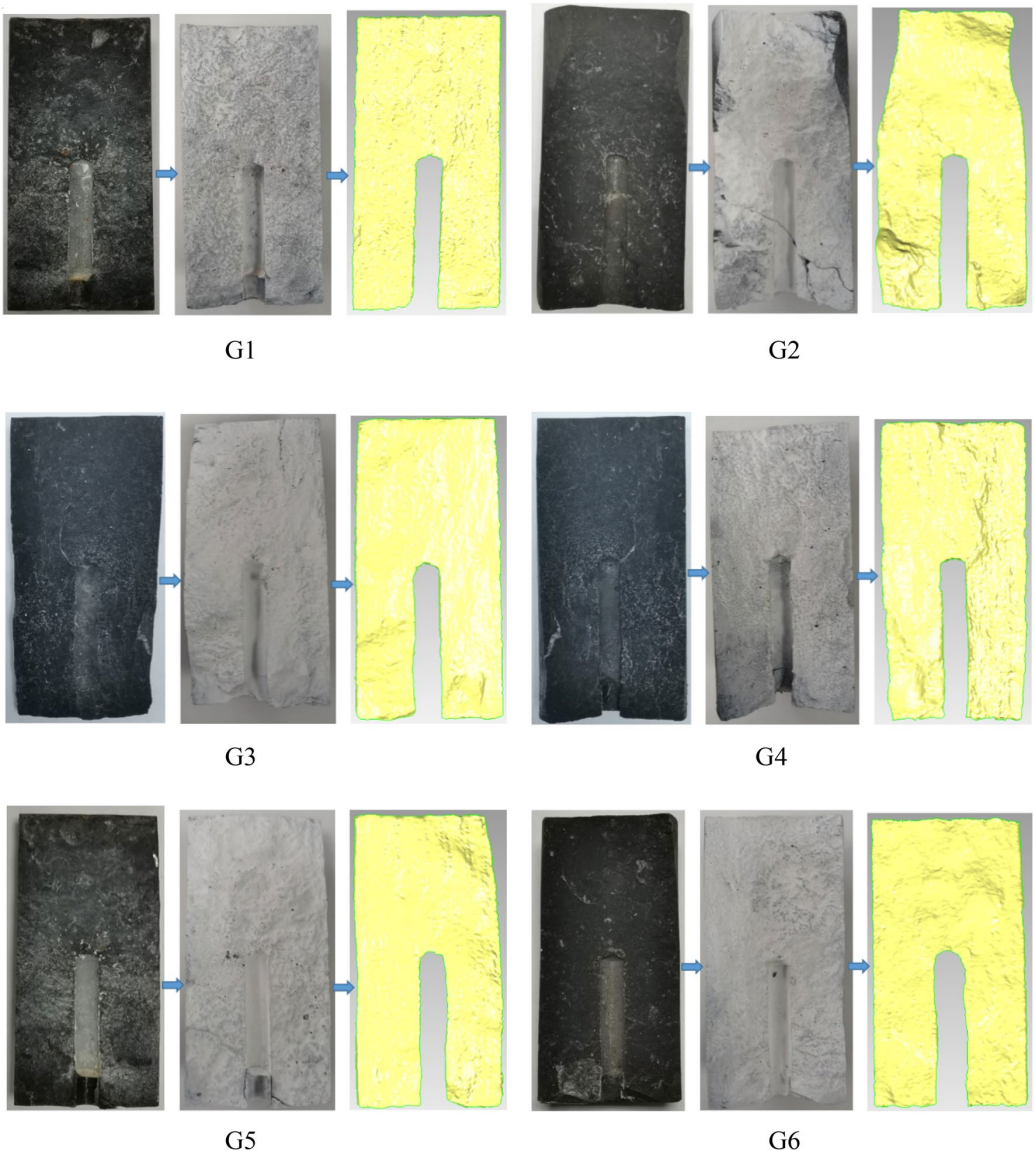
Crack surface morphology of each specimen.

In order to quantitatively analyze the roughness of the fracture surface after fracturing, first use three-dimensional scanner to scan the fracture three-dimensionally, and then use Geomagic Studio to reconstruct the fracture surface. The scanning and reconstruction process is shown in Fig. 8.

This paper introduces two parameters to quantitatively analyze the roughness of the crack surface, namely the area ratio (AR) and the standard deviation (SD). In order to evaluate the fracturing effect, He et al.^19^ used the specific surface to analyze the roughness of the fracture surface. At the same time, Liu^20^ and others used the standard deviation value to quantitatively characterize the crack surface roughness.

The ratio of the actual area of the crack surface to the projected area of the horizontal plane is used to define the area ratio (AR). The larger the ratio, the rougher the crack surface, as in formula (1) as follows:

1 $$ AR = A_{{1}} /A. $$

*A*_1_ and *A* are respectively expressed as the actual area of the crack surface and the horizontal projected area. In this experiment, *A*_1_ can be obtained by three-dimensional reconstruction, and A is the longitudinal cross-sectional area of the sample.

Standard deviation (SD) is a measure of the degree of deviation of the data value from the arithmetic mean, and its formula (2) is as follows:

2 $$ S = \sqrt {\frac{1}{N - 1}\sum\limits_{i = 1}^{N} {(X_{i} - X)^{2} } } . $$

**Figure 9:**
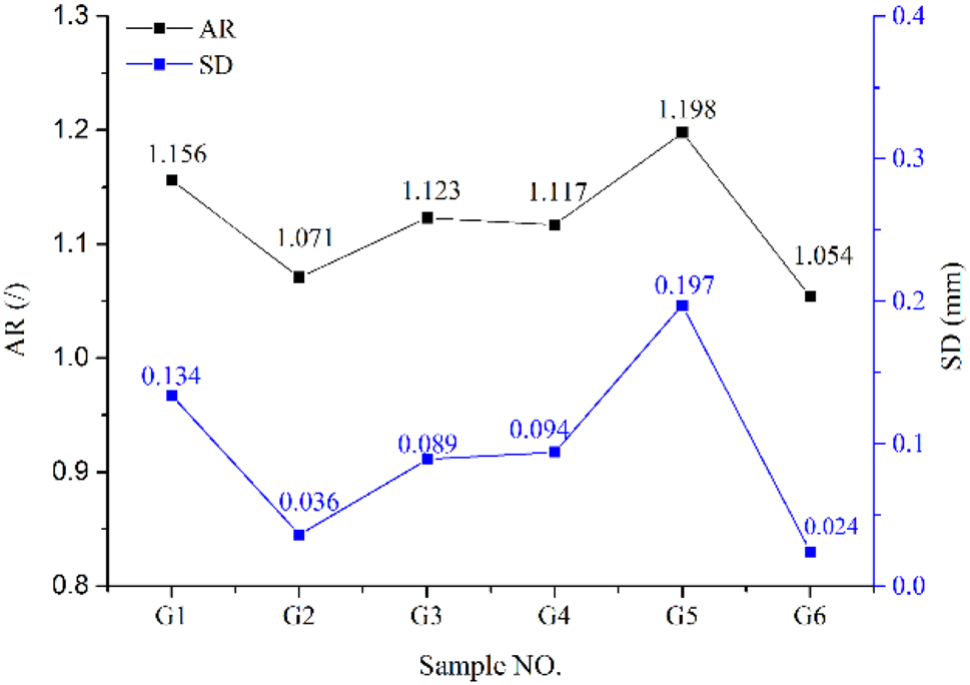
The relationship between the AR and SD of each test piece.

*N* represents the number of collected data, *X*_*i*_ represents each specific data value, and *X* represents the average value of the data. The standard deviation (SD) can be obtained by analyzing the Geomagic Studio software. Similarly, the larger the standard deviation value, the better the fracturing effect. The specific surface and standard deviation of the fracture surface of all shale specimens are shown in Fig. 9.

It can be analyzed from Fig. 9 that when the sand ratio is 10%, the roughness of the fracture surface of the shale specimen G5 reaches the maximum, that is, the specific surface (AR) is 1.198 and the standard deviation (SD) is 0.197 mm, indicating this When there is no proppant in the fracturing fluid, the fracture surface roughness of the shale specimen G6 is the smallest, and the specific surface (AR) and standard deviation (SD) both reach the minimum, respectively. 1.054 and 0.024 mm, indicating that the fracture surface under this test condition has the worst fracturing effect; from the comparison of shale specimens G1, G2, G3 and G4, it can be seen that as the confining pressure of the fracturing test increases and the fracturing fluid displacement. And the reduction of viscosity both lead to the reduction of the roughness of the fracture surface, indicating that both can cause the fracture surface to have a poor fracturing effect. For example, by comparing G1 and G2, it was found that the increase in confining pressure resulted in decrease in the surface roughness of the fracture; by comparing G1 and G3, it was found that the decrease in fracturing fluid viscosity led to decrease in fracture surface roughness of the fracture; by comparing G1 and G4, it was found that the decrease in fracturing fluid pump rate led to decrease in fracture surface roughness of the fracture; by comparing G1 and G5, it was found that the increase in fracturing fluid sand ratio led to increase in fracture surface roughness of the fracture; by comparing G1 and G6, it was found that no proppant resulted in the lowest fracture roughness, and illustrated the importance of proppant.

## Conclusion and discussion

The paper was mainly based on the hydraulic sand fracturing test under different conditions. It mainly studied the effects of different confining pressures, fracturing fluid displacement and viscosity, and different sand ratios, and finally compared it with the fracturing test without proppant. The importance of proppant in hydraulic sand fracturing was analyzed from the aspects of acoustic emission energy before and after fracture, positioning events, and fracture surface morphology. According to the experimental research results of the paper, the main conclusions can be shown as follows:

1. When hydraulic fracturing tests are carried out under different test conditions, the energy released by shale specimens before and after fracturing is different. The energy ratio is the largest when the sanding ratio is large, but the energy ratio obtained when hydraulic fracturing is performed without proppant is the smallest, and increasing the confining pressure, as well as reducing the displacement and viscosity of the fracturing fluid will cause the energy ratio to decrease. From the perspective of acoustic emission energy, it shows that proppant plays an important role in the generation of fractures during hydraulic sand fracturing.
2. Based on the number of acoustic emission positioning events before and after the fracture during hydraulic sand fracturing of shale specimens and the size of the distribution area for analysis and research. During the fracturing test, the increase in the sand ratio caused the maximum number of acoustic emission events after the specimen ruptured, and the total number of positioning events also reached the maximum; however, the number of shale specimens located before the rupture when the confining pressure increases The number of positioning events is the largest, but the total number of positioning events is smaller than that when the sanding ratio is increased; when there is no proppant hydraulic fracturing, the number of positioning of the specimen before and after the fracture and the total number both reach the minimum, indicating that the proppant is in the hydraulic sanding It can play an important role in the crack test.
3. According to the fracture surface morphology after three-dimensional scanning, from the two basic parameters of specific surface and standard deviation, the fracturing effect of proppant produced by shale specimens under different schemes is analyzed. When the sand addition is relatively large The specific surface and standard deviation both reach the maximum value, indicating that the fracture surface roughness is the largest under this test condition and the fracturing effect is the best.

## Data Availability

The data that support the findings of this study are available on request from the corresponding author.
